# Risk Compounds, Preclinical Toxicity Evaluation, and Potential Mechanisms of Chinese Materia Medica–Induced Cardiotoxicity

**DOI:** 10.3389/fphar.2021.578796

**Published:** 2021-03-30

**Authors:** Jie Zhou, Fu Peng, Xiaoyu Cao, Xiaofang Xie, Dayi Chen, Lian Yang, Chaolong Rao, Cheng Peng, Xiaoqi Pan

**Affiliations:** ^1^ State Key Laboratory of Southwestern Chinese Medicine Resources, Chengdu University of Traditional Chinese Medicine, Chengdu, China; ^2^ School of Pharmacy and School of Public Health, Chengdu University of Traditional Chinese Medicine, Chengdu, China; ^3^ Department of Pharmacy, Affiliated Hospital of Southwest Medical University, Luzhou, China; ^4^ West China School of Pharmacy, State Key Laboratory of Biotherapy, West China Hospital, Sichuan University, Chengdu, China

**Keywords:** Chinese materia medica, cardiotoxicity, risk compounds, preclinical toxicity evaluation, potential mechanisms

## Abstract

Chinese materia medica (CMM) has been applied for the prevention and treatment of diseases for thousands of years. However, arrhythmia, myocardial ischemia, heart failure, and other cardiac adverse reactions during CMM application were gradually reported. CMM-induced cardiotoxicity has aroused widespread attention. Our review aimed to summarize the risk compounds, preclinical toxicity evaluation, and potential mechanisms of CMM-induced cardiotoxicity. All relevant articles published on the PubMed, Embase, and China National Knowledge Infrastructure (CNKI) databases for the latest twenty years were searched and manually extracted. The risk substances of CMM-induced cardiotoxicity are relatively complex. A single CMM usually contains various risk compounds, and the same risk substance may exist in various CMM. The active and risk substances in CMM may be transformed into each other under different conditions, such as drug dosage, medication methods, and body status. Generally, the risk compounds of CMM-induced cardiotoxicity can be classified into alkaloids, terpenoids, steroids, heavy metals, organic acids, toxic proteins, and peptides. Traditional evaluation methods of chemical drug-induced cardiotoxicity primarily include cardiac function monitoring, endomyocardial biopsy, myocardial zymogram, and biomarker determination. In the preclinical stage, CMM-induced cardiotoxicity should be systematically evaluated at the overall, tissue, cellular, and molecular levels, including cardiac function, histopathology, cytology, myocardial zymogram, and biomarkers. Thanks to the development of systematic biology, the higher specificity and sensitivity of biomarkers, such as genes, proteins, and metabolic small molecules, are gradually applied for evaluating CMM-induced cardiotoxicity. Previous studies on the mechanisms of CMM-induced cardiotoxicity focused on a single drug, monomer or components of CMM. The interaction among ion homeostasis (sodium, potassium, and calcium ions), oxidative damage, mitochondrial injury, apoptosis and autophagy, and metabolic disturbance is involved in CMM-induced cardiotoxicity. Clarification on the risk compounds, preclinical toxicity evaluation, and potential mechanisms of CMM-induced cardiotoxicity must be beneficial to guide new CMM development and post-marketed CMM reevaluation.

## Introduction

Chinese materia medica (CMM) as an alternative therapy for disease treatment has become increasingly popular all over the world. A large number of clinical evidence has valued CMM, which is based on shifting compensatory homeostasis to the overall human body homeostasis, complementary to chemical medicine in the management of chronic disease (Fan et al., 2011). However, CMM formulations are often not subjected to premarket toxicity testing (Wang and Ren, 2002). Recently, more and more attention has been paid to the safety of CMM, including cardiotoxicity, hepatotoxicity, and nephrotoxicity of CMM. We previously reviewed some important information on CMM-induced liver injury (Pan et al., 2020). Drug-induced cardiotoxicity refers to the toxicity or negative effects of chemical drugs or CMM on the heart, including arrhythmia, myocardial ischemia, heart failure, and other cardiac adverse reactions (Li et al., 2015). A total of 81 drugs in the United States, Europe, and Asia from 1990 to 2013 were withdrawn from the market due to safety issues, including 16 drugs that caused arrhythmias (Li et al., 2016b). Chinese Adverse Drug Reaction Monitoring Report in 2018 showed that CMM accounted for 14.6% in adverse drug reactions/event reports and 8.7% in serious adverse reactions/event reports, and cardiovascular system damage caused by drugs accounted for 4.1% of all (National Medical Products Administration, 2019). General performances of drug-induced cardiotoxicity include electrocardiogram (ECG) abnormalities, myocardial infarction, impaired systolic and diastolic performance, functional remodeling and histopathological findings, and signs of apoptosis and degeneration. Acute cardiovascular injuries manifested as arterial hypertension, aortic dissection, arrhythmias, and myocardial ischemia. However, CMM-induced cardiotoxicity is easy to be ignored due to the lack of specific and detectable evaluation indicators.

At present, evaluation system on chemical drug-induced cardiotoxicity has been gradually established and improved, such as “The Clinical Evaluation of QT/QTc Interval Prolongation and Proarrhythmic Potential for Non-Antiarrhythmic Drugs” published by the International Conference on Harmonisation (ICH) in 2005, “Cardiovascular toxicity induced by chemotherapy, targeted agents and radiotherapy: ESMO Clinical Practice Guidelines” issued by the United States in 2012, and “Guidelines for Prevention and Treatment of Anthracycline-induced Cardiotoxicity” published by China in 2015 (Food and Drug Administration, 2005). However, the characteristics of multicomponent, multi-target, and multi-effect are involved in CMM-induced cardiotoxicity. There are not enough indicators of high specificity and accuracy to evaluate CMM-induced cardiotoxicity. Evaluation methods of cardiotoxicity caused by chemical drugs, such as anthracyclines, and chemotherapeutic and targeted drugs, are not fully applicable to those of CMM-induced cardiotoxicity. Therefore, evaluation strategies that meet the characteristics of CMM should be urgently established in the development and reevaluation of CMM. Previous studies mainly focused on chemical composition analysis, toxicity characteristics, mechanisms, and toxicokinetics on cardiotoxicity caused by single CMM or monomer compound of CMM. Our review aims to conclude these risk compounds, preclinical toxicity evaluation, and possible mechanisms of CMM-induced cardiotoxicity to guide clinical practice and promote the safety of drug application.

## Literature Retrieval Methods

A target search of available literature was performed on the PubMed, Embase, and China National Knowledge Infrastructure (CNKI) from January 1, 2000 to February 29, 2020 with the following key search terms: (“Chinese materia medica,” OR “traditional Chinese medicine,” OR “Chinese herbal medicine,” OR “Chinese medicine,” OR “medicinal herb,” OR “Chinese medicinal herb,” OR “Chinese herbs,” OR “herbal medicines,” OR “herbs”) AND (“cardiotoxicity,” OR “cardiac toxicity,” OR “cardiac poisonous,” OR “cardiac adverse effects,” OR “cardiac side effects,” OR “cardiac adverse events,” OR “cardiovascular events,” OR “cardiac damage,” OR “cardiac risk”). Individual scientific principles were applied to ensure the quality and relevance of searched articles. Two authors independently screened titles and abstracts of articles, excluding articles not relevant to the topic. Those studies on Chinese herbal compound prescriptions were also excluded, and only the relevant studies on CMM, their extracts, and ingredients were retained. The required articles must include a description of CMM-induced cardiotoxicity with scientific data, including full-length experimental articles, reviews, English-only abstracts, case reports, and conference papers.

## Risk Compounds of CMM-Induced Cardiotoxicity

The risk substances are the material basis for CMM-induced cardiotoxicity. Interestingly, the toxic material basis is closely related to the active material basis. Thus, misuse or overdose of CMM could trigger toxic effects instead of pharmacological action. According to different phytochemical structures, the risk compounds of CMM-induced cardiotoxicity are mainly classified into alkaloids, terpenoids, steroids, heavy metals, organic acids, and toxic proteins or peptides. Generally, more types of risk substances may be founded in a single CMM, while the same type of risk substance may exist in various CMM. Therefore, more attention should be paid to cardiotoxicity caused by CMM that contains these risk substances. The risk substances, toxicity evaluation indicators, and potential mechanisms of some common CMM-induced cardiac toxicity were concluded in Table 1.

**TABLE 1:** Risk substances, toxicity evaluation, and potential mechanisms of some representative CMM-induced cardiotoxicity *in vivo* and *in vitro*.

Risk substances			Toxicity evaluation		Potential mechanisms	References
Category	Risk compounds	CMM	Models and dosage	Measurement indicators		
Alkaloids	Aconitine	*Aconitum carmichaelii* Debeaux.	Primary culture of neonatal rat ventricular myocytes (NRVMs): 0–160 μmol/l for 7 days; AC-16 cells: 0–320 μmol/l for 24 h	Cell viability, mito-SOX (AC-16 cells), Notch1, NICD, HES1, c-Myc, CK2α, KDM5A, p300, RBP-J, HCN 4, *I*_*f*_ current, action potential, and beating rate	Involvement of Notch1/NICD/KDM5A/HCN4 toxicity pathway	Zhou et al. (2020)
		*Aconitum* sp.	Male Sprague–Dawley (SD) rats: 1.0 mg/kg/day by gavage for 7 days; H9c2 and rat primary cardiocyte cells: 0.5, 1, and 2 μmol/l for 24 h	ROS, mitochondria damage, TNF-α, FADD, Fas/Fas-L, cytochrome C, Bcl-2, caspase-3, caspase-8, RIP1, RIP3, MLKL, NLRP3, ASC, caspase-1, IL-1β, LC3-II (mitochondria), BNIP3, ULK1, LC3, and p62	Mitigation of BNIP3-dependent mitophagy and activation of the TNF-α-NLRP3 inflammatory pathways	Peng et al. (2020)
		*Aconitum carmichaelii* Debeaux.	Zebrafish embryo (cmlc2:eGFP): 1.87–30.0 μmol/l for 48 h; H9c2 cells: 0.75–6.0 mmol/l for 30 min	Survival rate, HR, the contraction of ventricles and atria, cell viability, intracellular Ca^2+^ concentrations, gene expression profile, cacna1c, RYR2, ATP2a2b, cTnC, Myh6, cTnT, p38, caspase-3, Bcl-2, and Bax	Ca^2+^ overload and cell apoptosis	Li et al. (2020)
	Hypaconitine	*Aconitum* sp.	Human embryonic kidney (HEK)-293 cells: 1, 3, 10, and 30 nmol/l; beagle dogs: 50, 150, and 450 μg/kg/day for 6 h	QTc interval and KCNH2 currents	Inhibition of KCNH2 potassium channels	Xie et al. (2015)
	Lappaconitine	*Aconitum* sp.	HEK-293 cells:10–100 μmol/l for 25–30 min	hH1 channels	Irreversible blockade of hH1 channels by binding to the site 2 receptor	Wright, (2001)
	Berberine	*Berberis* sp. or *Coptis* sp.	Guinea pigs: 27.1 mg/kg/day by gavage once; HEK-293 cells: 1 and 10 μmol/l for 24 h	HSP90, mature-155 kDa hERG, immature-135 kDa hERG, ATF6, calnexin, calreticulin, the colocalization between hERG and calnexin/calreticulin, hERG current, APD, and QTc interval	Induction of hERG channel deficiency by trafficking inhibition	Zhang et al. (2014)
	Evodiamine	*Evodia rutaecarpa* (*E. rutaecarpa*)	NRVMs: 31.3–250 μg/mL for 24 h; Zebrafish: 200–1,600 ng/mL for 24 h	Cell viability, LDH release, MDA, HR, heart malformation, pericardial edema, circulation abnormalities, thrombosis and hemorrhage, and SV-BA distance	Involvement of oxidative stress	Yang et al. (2017)
	Strychnine, brucine, and their N-oxide	*Strychnos nux-vomica* L. (Loganiaceae)	Male SD rats: 0.3 mg/kg and 0.6 mg/kg intravenously once; HEK 293 cells: 20–500 μmol/l for 10 min	QT interval and hERG channels	Inhibition of hERG channels	Yuan et al. (2018)
	Matrine, oxymatrine, cytisine, and sophocarpine	*Sophora tonkinensis* Gapnep. (*S. tonkinensis*)	hiPSC-CMs: 2, 10, and 50 μmol/l for 24 h	Cell viability, LDH, CK-MB, cTnI, SOD, GSH, ROS, MDA, and intracellular calcium	Induction of oxidative stress and disruption of calcium homeostasis	Wang et al. (2019b)
	Arecoline, guvacoline, and arecaidine	*Areca catechu* L.	Male Wistar rats: 4,500 mg/kg/day with arecae semen aqueous extract by gavage for 30 days	Body weight, CK, arachidonic acid, PGE2, l-tryptophan, linoleic acid, α-linolenic acid, oleic acid, palmitic acid, palmitoleic acid, and stearic acid	Disturbance of phospholipids, amino acids, and arachidonic acid metabolism	Lin et al. (2020)
Terpenoids	Triptolide	*Tripterygium wilfordii* Hook. F.	Male SD rats: 0.1 mg/kg by gavage for 14 days	Heart/body ratio, HR, myocardial fiber breakage, cardiomyocyte hypertrophy, cell gaps, nuclear dissolution, LDH, CK-MB, CAT, GSH, GSH-PX, plasma cTnI and GzmB, myocardial AhR, plasma AhR, CYP1 A1, and microRNAs	Changes in the expression of microRNAs and AhR	Wang et al. (2019c)
		*Tripterygium wilfordii* Hook. F.	H9c2 cell and primary rat cardiomyocyte: 0–640 nmol/l or 160 nmol/l for 2–24 h.; p53^−/−^ mice: 1.2 mg/kg intravenously once	Glucose (culture medium), ATP, LDH leakage, cell morphology, p65 (nuclei and cytoplasm), IKKβ, IκBα, N-p65/Histone3 p65, GLUT1, GLUT4, glucose uptake, TIGAR, Pgam2, and Pdk2	p53 mediates cardiac injuries *via* dysregulation of glucose uptake by blocking the IKKβ-NF-κB pathway	Xi, Zhang et al. (2020)
	Celastrol	*Tripterygium wilfordii* Hook. F.	Male Wistar rats: 0.5, 1, and 2 mg/kg by gavage for 7 days	Histopathological evaluation, MDA, SOD, valine, palmitic acid, sphingosine, lysophosphatidylcholine, 3-indolepropionic acid, 9-octadecenal, caspase-3, caspase-8, Bax, and Bcl-2	Palmitic acid–induced oxidative stress-regulated TNF/caspase axis	Liu, Zhang et al. (2019)
	Rhodojaponin I, II, and III	*Rhododendron molle G.* Don (Ericaceae)	Male SD rats: 21.44 and 112.56 mg/kg with *Rhododendri Mollis Flos* extract by gavage once	HR, LVSP, LVDP, maximum rate of developed left ventricular pressure (dP/dtm), maximum rate of decreased left ventricular pressure (-dP/dtm), ST-segment, LDH, CK-MB, and AST		Dong et al. (2014)
Steroids	Oleandrin and other cardiac glycosides	*Nerium oleander* L.	Male guinea pigs: 150 and 300 mg/kg/day with hydroalcoholic extract of oleander by gavage once; ventricular myocytes: 0.3 and 10 mg/mL with hydroalcoholic extract of oleander	ECG, mitochondrial structure, electron density, cardiac fiber, cardiac excitability, global Ca^2+^ transients, and Na^+^/K^+^-pump current	Inhibition of Na^+^/K^+^- pump, mitochondrial damage, and disturbance of calcium homeostasis	Botelho, Santos-Miranda et al. (2017)
	Periplocin	*Periploca sepium* Bge.	Neonatal rat cardiomyocytes: 0.2 and 0.4 mmol/l for 24 h	Tryptophan, carnitine, acetylcarnitine, citric acid, glutamic acid, pyroglutamic acid, leucine, pantothenic acid, indoleacrylic acid, proline, and lysophosphatidylcholine	Disruption of amino acid metabolism, energy metabolism, and sphingolipid metabolism	Li et al. (2016a)
	Bufo steroids	*Bufo bufo gargarizans* Cantor*; Bufo melanostictus* Schneider	Male SD rats: 100, 200, and 400 mg/kg by gavage for 48 h	HR, ST-segment, CK, CK-MB, ALT, AST, IL-6, IL-1β, TNF-α, MDA, SOD, CAT, GSH, GPX,TXNIP, NF-κB p65, IκBα, IKKα, IKKβ, ERK, JNK, and p38	Promotion of inflammatory response through the TXNIP/TRX/NF-κB and MAPK/NF-κB pathways	Bi et al. (2016)
	Bufalin	*Bufo bufo gargarizans* Cantor*; Bufo melanostictus* Schneider	Neonatal cardiac myocytes and adult rat cardiomyocytes: 40 and 400 ng/mL Chan Su extract, 4 and 20 ng/mL bufalin	Calcium transients, intracellular calcium, and Na^+^-K^+^ ATPase	Blockage of Na^+^- K^+^-ATPases and disturbance of calcium homeostasis	Roger et al. (2002)
Others	Arsenic trioxide	*Arsenolite*	HEK-293 cells and NRVMs: 3 μmol/l for 24 h	*I*_*Ca,L*_, APD, hERG channel, and Cav-1	Involvement of Cav-1 expression and promotion of hERG degradation	Yan et al. (2017)
		*Arsenolite*	Male BALB/c mice: 1 mg/kg/day via the tail vein for 2 weeks; ARVMs: 100 μmol/l for 20 min	Cardiomyocyte contractile function, intracellular Ca^2+^ transients, SERCA activity, SERCA2a, NCX, PLB, CaMKII, GRP78, PERK, eIf2α, IRE1, ATF6, CHOP, and caspase-12	Disturbance of Ca^2+^ homeostasis and ER stress-associated apoptosis	Zhang et al. (2017)
		*Arsenolite*	BALB/c mice: 1, 2, and 4 mg/kg/d *via* i.p. for 3, 7, and 14 days, H9c2 cell:0–10 µnol/l for 24 h	Mitochondrial structure, mPTP opening, ROS, ATP content, PGC-1α, DRP1, MFN1, MFN2, and OPA1	Cardiac mitochondrial damage and impaired energy metabolism	Zhang et al. (2018a)
	Aristolochic acid	*Aristolochia* sp.; *Asarum* L.	Zebrafish embryos: 1, 5, 10, and 20 μmol/l for 2–48 h	HR, cardiac phenotypes, COX-2, IL-1β, serum amyloid α, CCAAT/enhancer-binding protein B (C/EBPB), and C/EBPG	Induction of inflammatory response	Huang et al. (2007)
	Calcium oxalate needle crystal and lectin protein	*Pinellia ternata* (Thunb.) Breit.	SD rats: 3 g/kg/day with *Pinelliae Rhizoma* and its processed products by gavage for 14 days	Body weight, CK, CK-MB, LDH, histopathological evaluation, proline, leucine, tyrosine, saccharopine, 5-HT, dihydrouracil, KMTB, kynurenine, dhS1P, p-aminobenzoic acid, TGF-β1, mTOR, and MDA	Induction of inflammatory response through inhibiting mTOR signaling and activating the TGF-β pathway	Su et al. (2016)
	Aag-FG_50_ (scorpion toxin)	*Androctonus australis hector*	Mongrel dogs: 0.05 mg/kg of the purified venom toxic fraction by intravenous injection once	HR, mean arterial pressure, pulmonary artery occluded pressure, cardiac output, stroke volume, systemic vascular resistances, serum lactate, epinephrine, norepinephrine, neuropeptide Y, endothelin-1, and atrial natriuretic peptide	Disturbance of hemodynamic by excessive catecholamines release	Nouira et al. (2005)

### Alkaloids

#### Diterpene Alkaloids

Alkaloids are the most common risk compounds in CMM-induced cardiotoxicity. Diterpenoid alkaloids are the largest category of alkaloids that lead to cardiac toxicity, which can be found in aconitum plants, such as *Radix Aconiti lateralis Preparata*, *Radix Aconiti*, *Radix Aconiti Kusnezoffii*, and *Radix Aconiti Brachypodi*, as well as their raw and processed products. According to different phytochemical structure, aconite alkaloids mainly include C-18, C-19, and C-20 types of diterpenoid alkaloids. C-19 diterpenoid alkaloids are the most common and most toxic ingredients, including aconitine, hypoaconitine, mesoaconitine, benzoyl aconitine, benzoyl hypoaconitine, and benzoyl mesoaconitine (Nyirimigabo et al., 2015). Aconitine and its analogs have been demonstrated to induce lethal malignant ventricular arrhythmias or acute myocardial infarction (Sun et al., 2014; Xie et al., 2015). Aconitine has even been developed into experimental tools to establish cardiac arrhythmic models in cardiovascular drug-related researches (Ma et al., 2018). In addition, other diterpenoid alkaloids, such as lappaconitine, ranaconitine, delvestidine, denudatine, secokaraconitine, and brachyaconitine, have also been shown to have obvious cardiotoxicity (Wang et al., 2014a; Nie et al., 2017).

#### Isoquinoline Alkaloids

Berberine, a class of isoquinoline alkaloids, is considered to be the major risk substance in various CMM, such as *Coptis chinensis* and *Cortex Phellodendri Chinensis*. It has been reported that berberine acutely inhibits human ether-a-go-go-related gene (hERG) currents and prolongs action potential duration in *Xenopus* oocytes (Li et al., 2001). Studies *in vivo* showed that high concentrations of alkaloids, including berberine, coptisine, palmatine, and jatrorrhizine, were found in heart tissues of mice. Results of *in vitro* experiments also suggested that berberine, coptisine, palmatine, and jatrorrhizine presented cytotoxicity in dose- and time-dependent manners in H9c2 cells (Ma et al., 2010). Some studies demonstrated that berberine, berbamine, palmatine, and oxyberberine could result in cardiac arrest and arrhythmia in a time- and dose-dependent manner (Zhang et al., 2018b). In addition to berberine and coptis alkaloids, Papaveraceae plant *Chelidonium majus* also contains other isoquinine alkaloids, such as sanguinarine, chelidonine, and chelerythrine. Direct effects of sanguinarine on the heart may be responsible for cardiac arrest and death (Hu et al., 2005). The concentration- and time-dependent apoptoses to necrosis were observed during chelerythrine administration in rat neonatal ventricular cardiomyocytes or human pluripotent stem cell–derived cardiomyocytes (Mioulane et al., 2012).

#### Indoles Alkaloids

Indoles alkaloids, such as strychnine and brucine isolated from *Strychnos nux-vomica* L., have been shown to be cardiotoxic. An automatic patch-clamp system was established to screen and evaluate strychnine for cardiotoxicity. Results showed that strychnine inhibited hERG, increased QT interval, and decreased heart rate (HR) in isolated rat hearts (Wang et al., 2018). Both strychnine and brucine were shown to exhibit the voltage-dependent inhibition of hERG channels (Yuan et al., 2018). Moreover, other indole alkaloids, such as evodiamine, vinblastine, and vincristine, are specified as the risk substances of *Evodiae fructus* and *Catharanthus roseus*, respectively. Results indicated that evodiamine led to cardiovascular side effects in primary cultured neonatal rat cardiomyocytes and zebrafish (Yang et al., 2017). Tubulin-binding drugs containing vinblastine and vincristine presented specific toxicity through pathologic, immunological, and transcriptomic analyses in cardiac endothelial cells (Mikaelian et al., 2010).

#### Organic Amine Alkaloids

Organic amine alkaloids, such as ephedrine and colchicines, have been gradually proved to be cardiotoxic. The study indicated that ephedrine isolated from *Ephedra sinica Stapf* could trigger a range of cardiovascular toxicities, including myocarditis, arrhythmias, myocardial infarction, cardiac arrest, heart failure, and sudden death (Samenuk et al., 2002; Adamson et al., 2004; Naik and Freudenberger, 2004; Nyska et al., 2005). Colchicine derived from *Colchicum autumnale* has been proved to impair impulse formation and conduction (Tochinai et al., 2014). Another study suggested that colchicine could directly damage cardiac endothelial cells to induce cardiotoxicity (Mikaelian et al., 2010).

### Terpenoids

Terpenoids, such as triptolide, celastrol, and rhodojaponin, are other important compounds of CMM-induced cardiotoxicity. Triptolide, a highly promising diterpenoid triepoxide extracted from *Tripterygium wilfordii*, has been widely reported to elicit chest distress, cardiopalmus, bradyarrhythmia, and even cardiogenic shock (Huang et al., 2009). Some studies demonstrated that triptolide induced cardiomyocyte damage and cell apoptosis (Li et al., 2014; Zhou et al., 2015). Celastrol, a cell-permeable dienonephenolic triterpene compound, was also derived from *Tripterygium wilfordii*. Celastrol was revealed to have potent inhibitory activity on potassium channels, thereby inducing QT interval prolongation (Sun et al., 2006). Various toxic symptoms of zebrafish embryos were caused by celastrol, including appearance of heart linearization, heart membrane hemorrhage, and hemocyte accumulation in the cardiac region (Wang et al., 2009). Besides, grayanane diterpenoids, the main active component extracted from the fruits of *Rhododendron molle*, were related to cardiotoxicity (Zhou et al., 2014). A study on the toxicological effects of rhodojaponins demonstrated that myocardial injury was elicited by *Rhododendron molle* extract (Dong et al., 2014).

### Steroids

Steroids, such as cardiac glycosides, cardenolides, and steroid saponins, are also revealed to induce cardiotoxicity. Cardiac glycosides are the largest category of steroids, such as digitalis derivatives, oleandrine, and periplocin. Digitalis derivatives have been known as a representative compound of cardiac glycosides. Several studies showed that digitalis derivatives presented arrhythmogenic effects on the heart (Demiryurek et al., 2002; Demiryürek and Demiryürek, 2005; Ramirez-Ortega et al., 2007). Arrhythmias and electrical conduction disturbances promoted by digitalis derivatives are related to cardiomyocyte dysfunction (Botelho et al., 2017). Oleandrin, a cardiac glycoside derived from the leaves of *Nerium indicum*, has been shown to induce myocardial damage *in vivo* and *in vitro* (Poindexter et al., 2007; Zhou et al., 2019). Periplocin, the main substance in *Periploca sepium Bunge*, easily triggered cardiotoxicity because of improper application (Demiryürek and Demiryürek, 2005). Moreover, cardenolide is another representative steroid compound of CMM. Bufadienolides, including resibufogenin, arenobufagin, bufalin, and cinobufagin, are natural cardenolides, which are also the major pharmacological constituents of cinobufagin venom toad. Induced resibufogenin delayed after depolarization and triggered arrhythmias in cardiac fiber at high concentrations *in vitro* and *in vivo* (Xie et al., 2002). Arenobufagin was also reported to induce obvious myocardial damage (Deng et al., 2017; Hou et al., 2018). Steroid saponins in some CMM have also been shown to be cardiotoxic. For example, ophiopogonin D, a steroidal saponin derived from *Ophiopogon japonicus*, presented toxic effects on cardiomyocytes (Ren et al., 2017; Wang et al., 2019a). Ginsenosides Rb1 and Re were found to inhibit cardiac contraction in ventricular myocytes (Scott et al., 2001).

### Other Risk Compounds

Heavy metals, organic acids, toxic proteins, and peptides may also induce some cardiac adverse reactions. As_2_O_3_, the main ingredient of the highly toxic substance arsenic, has been proved to have a variety of adverse cardiac reactions, such as cardiac arrhythmia, ventricular tachycardia, or even sudden cardiac death (Barbey et al., 2003; Balakumar and Kaur, 2009; Zhang et al., 2013b). As_2_O_3_ was also shown to lead to human cardiac developmental toxicity (Bao et al., 2019). Aristolochic acid derived from *Aristolochia debilis*, *Aristolochia manshuriensis*, or *Asarum sieboldii* has been found to specifically trigger heart defects in zebrafish embryos (Huang et al., 2007; Shi et al., 2017). Toxic proteins and peptides mainly derived from some plants or animals have been widely reported to exert cardiac toxicity effects. Scorpion toxins, such as BmSKTx1, BmBKTx1, BmTX1, BmTX2, and BmP09 identified from buthotoxin, are the main substances that are responsible for cardiotoxicity (Xu et al., 2004a; Xu et al., 2004b; Yao et al., 2005; Quintero-Hernandez et al., 2013). Centipede toxin, a type of polypeptide toxin purified from *Scolopendra subspinipes*, including SSD609 and SsTx, can induce cardiovascular system disorders (Sun et al., 2015; Luo et al., 2018).

## Preclinical Evaluation of CMM-Induced Cardiotoxicity

The early identification of CMM-induced cardiotoxicity is quite difficult due to the lack of specific evaluation methods. Evaluation of chemical drug-induced cardiotoxicity can provide a reference for CMM-induced cardiotoxicity. CMM-induced cardiotoxicity presents a variety of manifestations, including abnormal ECG, myocardial infarction, impaired systolic and diastolic performance, functional remodeling and histopathological changes, and signs of myocardial apoptosis and degeneration. Thus, CMM-induced cardiotoxicity should be evaluated at the overall, tissue, cellular, and molecular levels, including cardiac function, morphological alteration in cardiac muscle tissue, cell or organelle structures and function, myocardial zymogram, and biomarkers.

### Cardiac Function

At present, various clinical examination methods can be used to evaluate the effects of CMM on cardiac function. Generally, the evaluation of cardiac function involves HR, blood pressure, hemodynamic parameters, ECG, echocardiography, and magnetic resonance imaging (MRI) examination. ECG, the most common technique for monitoring drug-induced cardiotoxicity, can record bioelectrical changes during the occurrence, spread, and recovery of cardiac excitability. Various arrhythmias, ventricular atrial hypertrophy, myocardial infarction, and myocardial ischemia can be identified by ECG monitoring. Electrophysiological alterations of drug-induced cardiotoxicity mainly involve the prolongation of QT/QTc interval, action potential duration (APD), and ST-segment alteration. QT and QTc interval can be prolonged 12–24 h after myocardial infarction. QT interval prolongation is also significantly associated with arrhythmia, especially ventricular arrhythmia and torsades de pointes (TdP) (Halkin et al., 2001). Similarly, the prolongation of APD-induced arrhythmia, following ventricular hypertrophy, can also lead to impaired myocardial relaxation and contractile function (Odening et al., 2013). The risk substances of cardiotoxicity caused by CMM, such as arsenic trioxide, berberine, hypaconitine, aconitine, and strychnine, usually induce the prolongation of APD and QT/QTc interval, thus causing various arrhythmias, abnormal cardiac systolic, and other adverse reactions (Wang et al., 2008; Chen et al., 2010; Zhang et al., 2014; Xie et al., 2015; Wang et al., 2018). Aconitine not only triggered sinus rhythm with low-amplitude P waves and junctional rhythm conducting with narrow QRS complex but also presented diffuse ST-segment depression and the prolongation of QT interval (Karturi et al., 2016). Aconitine and *Venenum Bufonis* disrupted heart rhythm, influenced diastolic function, and significantly promoted ST-segment elevation (Sun et al., 2014; Bi et al., 2016). *Rhododendron molle* extract–treated rats also showed an ST-segment elevation, following acute ischemic tissue injury (Dong et al., 2014). Intravenous injection with toad extract provoked supraventricular arrhythmia (Kostakis and Byard, 2009). Therefore, ECG is one of the effective indicators for identifying the selective toxicity of CMM to the heart. ECG abnormality induced by CMM is able to reflect myocardial diseases, such as arrhythmia, conduction block, and myocardial ischemia. However, ECG is difficult to detect the early cardiotoxicity induced by CMM if left ventricular ejection fraction (LVEF) is normal. Thus, ECG should be combined with other indicators to systematically evaluate the cardiotoxicity of CMM.

### Histopathology

Histopathological examination, the highest standard for determining chemical drug-induced cardiotoxicity, can also be applied for evaluating CMM-induced cardiotoxicity. Inflammatory cell infiltration, myocardial rupture, and hemorrhage were clearly observed after a high dose *Aconitum carmichaelii* Debx. treatment in the cardiac muscle tissue of mice (Cai et al., 2013). Myocardial fiber breakage, cell swelling, and interstitial congestion were found in a larger number of mice exposed to triptolide (Yang et al., 2016). The dilated intercellular spaces were observed in severely damaged myocardium of *Venenum Bufonis*–treated rats, which were accompanied by abundant eosinophilic cytoplasm, obvious inflammatory cell infiltrations, and moderate piecemeal necrosis (Dong et al., 2015). Zebrafish has been widely applied to study the cardiotoxicity of new drugs with the advantages of small size and transparent body. Morphological abnormalities in zebrafish were observed and quantitatively assessed, including heart malformation, pericardial edema, circulation abnormalities, thrombosis, and hemorrhage (Yang et al., 2017).

### Cell Morphology

To elucidate the potential mechanism, evaluation on CMM-induced cardiotoxicity should be deepened to the cellular and molecular levels. As an alternative to traditional animal model assessment, cytological evaluation *in vitro* has significant advantages in identifying early, mild, and potential toxicity of CMM (Chen et al., 2017). Toxic effects of CMM on myocardial cells could be visually determined through cell morphology observation. At present, the inverted phase contrast microscopy, fluorescence microscopy, transmission electron microscopy, and laser scanning confocal microscopy provide convenience for observing the microstructure and submicroscopic structures in cells. A study utilized a fluorescent microscope to observe the effects of As_2_O_3_ on bone marrow mesenchymal stem cells (BMSCs) through Hoechst 33,342 staining (Cai et al., 2010). Results demonstrated that As_2_O_3_ induced abnormal morphological features, such as cellular shrinkage and broken nuclei in BMSCs. In addition, hyperchromatic and dense fluorescent particles within the massive apoptotic nuclear cytoplasm were observed under fluorescence microscopy after aconitine administration (Ma et al., 2018). Beating rhythm, sarcomere shortening, and Ca^2+^ transients of ventricular myocytes were assessed during aconitine-induced cardiotoxicity using video-based sarcomere contractility and calcium recording module (Sun et al., 2014). One study evaluated the effects of aconitine on electrophysiological changes of ventricular myocytes by the patch-clamp recording technique. Results showed that APD for both 50 and 90% repolarization was significantly prolonged by aconitine at a dose of 1 μmol/l (Zhou et al., 2013). Another study assessed cell shortening of ventricular myocytes by a video-based edge-detection system. Results suggested that cinobufagin decreased the maximum cell shortening along with the decrease in Ca^2+^ transients (Liu et al., 2014).

### Myocardial Zymogram

Myocardial enzymes widely distributed in cardiomyocytes will be released into the blood circulation if myocardial cells are damaged. These myocardial enzymes, including creatine kinases (CK), creatine kinase isoenzymes (CK-MB), lactic dehydrogenase (LDH), aspartate aminotransferases (AST), and α-hydroxybutyrate acid dehydrogenases (α-HBDH), are able to reflect myocardial ischemic necrosis or cell membrane permeability, which together form myocardial zymogram to evaluate CMM-induced cardiotoxicity. For example, studies showed that As_2_O_3_ could increase serum AST, CK, CK-MB, LDH, and α-HBDH levels in chicken, which were related to myocardial injury (Zhang et al., 2013a; Li et al., 2017). *Rhododendri Mollis Flos*–induced cardiotoxicity was evaluated by measuring the plasma levels of LDH, CK-MB, and AST (Dong et al., 2014). After SD rats were gavaged with *Tripterygium wilfordii* water decoction for 14 consecutive days, LDH and CK-MB levels in serum were significantly reduced, indicating that these enzymes were expected to early predict and identify CMM-induced cardiotoxicity (Wang et al., 2016a). LDH, AST, CK, and α-HBDH have poor specificity for evaluating drug-induced cardiotoxicity, while CK-MB has high sensitivity and specificity for diagnosing acute myocardial infarction. Studies revealed that the cardiotoxicity of anthracyclines could be manifested as a significant increase in CK-MB, which could be used as an indicator for the auxiliary diagnosis of heart damage (Chen et al., 2016). CK-MB detection also has a high application value in neonatal myocardial injury after asphyxia, which can provide a strong reference for clinical diagnosis (Huang, 2019). CK-MB is also more widely used in clinical monitoring of cardiotoxicity caused by drugs than brain natriuretic peptide (BNP) and N-terminal brain natriuretic peptide precursor (NT-proBNP). Clinical observation of 112 cancer patients treated with paclitaxel found that the incidences of paclitaxel-induced arrhythmia and myocardial zymogram abnormality were, respectively, 26.8 and 44.6% in the normal group, while that in the experimental group were, respectively, as high as 37.3 and 68.4%, indicating that myocardial zymogram was more sensitive to CMM-induced cardiac toxicity than arrhythmia (Lu and Chen, 2010).

### Biomarkers

#### Proteins or Polypeptides

Some novel cardiac dysfunction markers, such as natriuretic peptides and myocardial proteins, have also been gradually applied to assess the cardiotoxicity of CMM. These natriuretic peptides, including atrial natriuretic peptide (ANP), BNP, and NT-proBNP, regulate the homeostasis of circulating volume and blood pressure, which have been widely used as markers for heart failure (Namdari et al., 2016; Maisel et al., 2018). Aristolochic acid upregulated the expression of BNP and ANP during the induction of zebrafish heart failure model (Shi et al., 2017). Troponins, including troponin T (cTnT) and troponin I (cTnI), are highly specific and sensitive to myocardial injury. Serum levels of cTnI and cTnT were significantly elevated during myocardial injury or infarction and heart failure (Panteghini, 2004; Horacek et al., 2007). cTnI and plasma granulase B in SD rats were significantly increased by water decoction of *Tripterygium wilfordii*. Studies on the cardiotoxicity of triptolide and *Radix Aconiti* extract revealed that serum cTnI level increased after administration (Lin et al., 2011; Xi et al., 2018). Acute aconitine intoxication manifested as remarkable elevation in serum CK, CK-MB, and cTnI levels (Lin et al., 2011). Myoglobin can reflect the extent of myocardial injury and the degree of myocardial necrosis. Myoglobin significantly increased in the early stage of myocardial injury before myocardial enzyme production, indicating that myoglobin is more sensitive than serum CK-MB for CMM-induced cardiotoxicity. However, myoglobin should be combined with troponins as biomarkers to evaluate the cardiac toxicity of drugs because of the low expression of myoglobin in myocardium. Although natriuretic peptides and troponins are recommended for assessing myocardial injuries with the advantages of higher specificity and sensitivity than myocardial enzymes, they are still less used for studying CMM-induced cardiotoxicity in clinical application. Cardiac fatty acid-binding protein (FABP) in the blood is better than CK and cTnI in the diagnosis of early myocardial injury (especially ≤ 3 h), which has high specificity and sensitivity for predicting CMM-induced myocardial damage (Wang et al., 2014b).

#### Metabolic Small Molecules

Some studies showed that citric acid, glutathione (GSH), phosphatidylcholine, and uric acid in serum could be used as biological indicators for detecting CMM-induced cardiotoxicity. Once the cardiovascular system is damaged, citric acid in serum decreases, while sugar and amino acid levels increase. The removal of intracellular reactive oxygen species (ROS) is usually accompanied by a reduction in GSH. Phosphatidylcholine is converted to arachidonic acid under the catalysis of phospholipase A2. Arachidonic acid is further converted into thromboxane A2 and thromboxane B2 by cycloprostaglandin H2 and oxygenase prostaglandin G2, thus inducing a series of cardiovascular diseases, such as blood stasis and thrombus. Therefore, phosphatidylcholine content sharply decreases under certain pathological conditions. A study found that the contents of endogenous biomarkers GSH, phosphatidylcholine, and citric acid in rat serum decreased, while ascorbic acid, uric acid, d-galactose, tryptophan, and l-phenylalanine levels increased after combined administration with ginseng and aconite (He et al., 2015). Once myocardium is short of blood or energy, ATP will be rapidly degraded to hypoxanthine and xanthine, and the final metabolite uric acid will be produced in large quantities, in which a large amount of ROS is generated, suggesting that uric acid level can reflect cardiovascular system damage. More than 20 biomarkers in serum and myocardial tissue of rats were detected after continuous gavage with *Asarum sieboldii* for 28 days by the nuclear magnetic resonance hydrogen spectroscopy (1HNMR), which was represented by a significant increase in lipid, lactic acid, alanine, pyruvic acid, dimethylglycine, β-glucose, and α-glucose contents, and a significant decrease in glutamic acid and tyrosine contents (You, 2016). At present, carbohydrate metabolites (α-ketoglutarate, malic acid, glucose, fructose, and succinic acid), sphingolipids (sphingosine, dihydrosphingosine, and phytosphingosine), organic acids (citric acid, pantothenic acid, and taurine), and carnitines (L-palmitoylcarnitine, L-acetyl carnitine, and isobutyryl-l-carnitine) can also be used as biomarkers for identifying CMM-induced cardiotoxicity (Qi et al., 2014). Studies showed that 11 biomarkers significantly decreased following the increased doses of periplocin extracted from *Periploca sepium Bunge*, including carnitine, acetylcarnitine, lysophosphatidylcholine, proline, glutamic acid, leucine, pantothenic acid, tryptophan, indole acrylic acid, and citric acid (Li et al., 2016a). These metabolites may affect the normal physiological function of the heart in terms of cell membrane stability, oxidative stress, mitochondrial damage, energy metabolism, cell homeostasis, and cell-to-cell signaling and interaction.

#### Genes

Omic technologies, especially genomics and transcriptomics, have gradually deepened in the field of drug toxicology, which provide new techniques for predicting new biomarkers to evaluate CMM-induced cardiotoxicity. Gene chip technology combined with real-time fluorescence quantitative analysis was applied to study acute toxicity of *Venenum Bufonis* on rat heart (Yang et al., 2011a). Results showed that *Venenum Bufonis* significantly altered the expression of Fxyd domain-containing ion transport regulator 3 (Fxyd3), Rho family GTPase 1 (Rnd1), ceruloplasmin, and Jun oncogene, which were involved in ion homeostasis, actin construction, and apoptosis. A study on the cardiotoxicity of aristolochic acid in zebrafish embryos revealed that a number of pro-inflammatory genes significantly increased, including COX-2, IL-1β, C/EBPB, C/EBPG, and SAA (Huang et al., 2007; Shi et al., 2017). In recent years, microRNAs have been linked to the cardiotoxicity of drugs, such as anthracyclines, bevacizumab, and cyclosporine A (Skala et al., 2019a). Some studies have found that the changes of some microRNAs are able to reflect the degree of myocardial injury in patients with acute myocardial infarction (AMI) (Yao, 2013). Studies suggested that As_2_O_3_ suppressed cardiac potassium channels *via* upregulating miR-21, miR-23a, miR-133, and miR-1 (Shan et al., 2013; Zhao et al., 2015). Although few studies about microRNAs are rarely involved in the cardiac toxicity of CMM, microRNAs have potential as early biomarkers and diagnostic tools for CMM-induced cardiotoxicity.

## Mechanisms of CMM-Induced Cardiotoxicity

**FIGURE 1:**
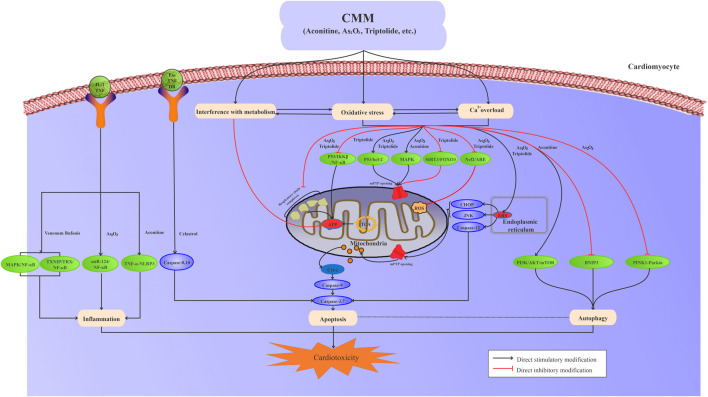
Interaction among oxidative stress, calcium overload, metabolic disturbance, mitochondrial damage, apoptosis, autophagy, and inflammation is involved in CMM-induced cardiotoxicity.

The risk compounds of CMM-induced cardiotoxicity directly or indirectly affect the structure and function of cardiomyocytes. Generally, multiple mechanisms interact with each other to induce cardiotoxicity, including ion homeostasis, oxidative stress and lipid peroxidation, mitochondrial damage, programmed cell death, inflammation, and metabolic disturbance (Figure 1).

### Interference With Ion Homeostasis

**FIGURE 2:**
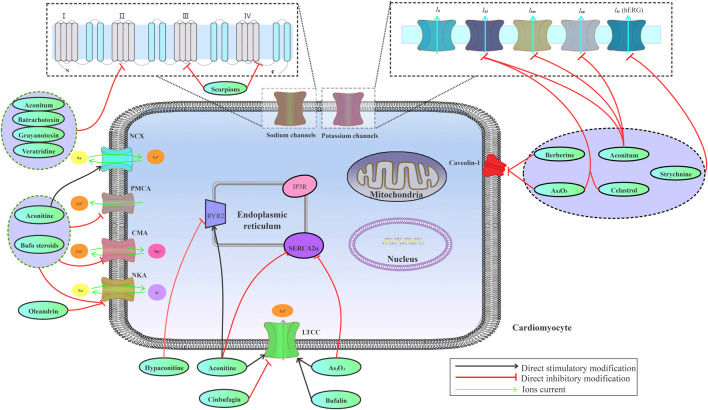
Effects of the risk compounds in CMM on cardiac sodium, potassium, and calcium channels.

Various ion channels, including sodium, potassium, and calcium channels, are highly expressed on the myocardial cell membrane, which work together to maintain ion homeostasis in cardiomyocytes. Ion channel abnormalities on cardiomyocytes may lead to alterations in electrophysiological activity of the heart, ultimately inducing arrhythmias. The imbalance among sodium, potassium, and calcium ions in myocardial cells contributes to CMM-induced cardiotoxicity (Figure 2).

#### Sodium Channels

Sodium channel affects cardiomyocyte excitability, conductivity, auto-rhythm, and excitatory contraction coupling. The risk compounds of CMM-induced cardiotoxicity, especially alkaloids, can modulate the gating, permeability, and selectivity of voltage-gated sodium channels (VGSCs) by binding to site receptors. For example, aconitine binds to the site 2 receptor on VGSCs, thereby causing kinetic shift of sodium channels to trigger a persistent activation of sodium channels (Rao and Sikdar, 2000; Amran et al., 2004; Tak et al., 2016). In contrast to aconitine, a sodium channel agonist, lappaconitine irreversibly blocked human heart sodium channels by binding to the site 2 receptor (Wright, 2001). Moreover, batrachotoxin, one of the main active ingredients of *Venenum Bufonis*, is considered as a full activator on sodium channels, while aconitine and its analogs are served as partial activators (Wang et al., 2007). Grayanotoxins are toxic diterpenoids present in leaves of *Rhododendron*, *Kalmia*, *Leucothoe*, and Ericaceae (Yang et al., 2011b). Grayanotoxins exert selective effects on voltage-dependent sodium channels by eliminating fast sodium inactivation and inducing a hyperpolarizing shift in voltage dependence of channel activation (Maejima et al., 2003). Veratridine, a steroid-derived alkaloid derived from rhizomes of *Veratrum album* or seeds of *Schoenocaulon officinale*, preferentially binds to the activated sodium channels, thus impeding inactivation and increasing cell excitability (Chevalier et al., 2014). However, toxins secreted from scorpions are currently classified into two categories based on physiological effects on channel gating and binding properties: α-toxins that inhibit the fast inactivation process of sodium channels by binding to the site 3 receptor, and β-toxins that shift the threshold of channel activation to the negative membrane potentials by binding to the site 4 receptor (Ortiz and Possani, 2018; Wang et al., 2019b). SCN5A that is highly expressed in human cardiac muscle cells is the important gene encoding myocardial sodium channels, which controls the excitatory conduction of cardiac muscle cells. Studies showed that both aconitine and hypaconitine were shown to enhance mRNA expression of the SCN5A ion channel gene (Liu, 2007; Li et al., 2011). Abnormality in SCN5A gene expression can lead to an increase in sodium influx, which is associated with a variety of arrhythmias induced by some toxic CMM (Korkmaz et al., 2013).

#### Potassium Channels

The delayed rectifying potassium current (*I*_*kr*_) channel protein on human cardiomyocytes is encoded by human hERG. Studies demonstrated that the loss of hERG function and the inhibition of *I*_*kr*_ caused by drugs prolonged QT interval and possibly induced TdP, thus leading to fatal arrhythmias (Liu et al., 2017). Some toxic CMM induce cardiotoxicity by directly inhibiting the expression of hERG channel protein or coding gene to suppress potassium channels. For instance, berberine blocked *I*_*k1*_, *I*_*k*_, and hERG channels expressed in *Xenopus* oocytes (Li et al., 2001). Diterpene alkaloids, such as aconitine and hypaconitine, induced QT interval prolongation through the inhibition of hERG potassium channels (Li et al., 2010; Xie et al., 2015; Kiss et al., 2017). Studies revealed that selectively targeting hERG channel contributes to the cardiotoxicity of strychnine (Wang et al., 2018). Celastrol inhibited the activities of hERG potassium channels, causal to QT interval prolongation (Sun et al., 2006). As_2_O_3_ induced expression deficiency of hERG potassium channel by decreasing specificity protein 1 (Sp1) level that upregulated hERG channel expression (Zhang et al., 2013b). MicroRNAs play a crucial role in regulating cardiac electrophysiological function by targeting potassium channel genes. Both miR-21 and miR-133 repressed the expression and transcription of hERG in As_2_O_3_-induced hERG inhibition. CMM also reduced *I*_*kr*_ through disrupting hERG trafficking without channel blockage. For example, berberine and arsenic induced hERG channel deficiency by inhibiting channel trafficking (Ficker et al., 2004; Eckhard Ficker and Peter Hawryluk, 2004). Unfolded protein response (UPR) was activated by channel trafficking inhibition, and endoplasmic reticulum (ER)-restricted hERG was ubiquitinated and degraded in lysosomes and proteasomes (Zhang et al., 2014). In addition, some CMM reduce *I*_*kr*_ *via* promoting hERG channel protein degradation, which involves the regulation of related genes, proteins, and signaling pathways. The integral membrane protein caveolin-1 is co-localized with hERG on the cell surface, which has been reported to be involved in the drug-induced reduction of hERG plasma-membrane expression (Lin et al., 2008). Berberine was proved to reduce hERG membrane stability by disrupting caveolin-1 (Rodriguez-Menchaca et al., 2006; Yan et al., 2015). Lysosomal and proteasome degradation of hERG on the plasma membrane were accelerated during As_2_O_3_-induced cardiotoxicity. Further research showed that As_2_O_3_ decreased the caveolin-1 level, which accounted for As_2_O_3_-triggered hERG degradation (Yan et al., 2017). Certainly, some CMM can also affect potassium channels by suppressing *I*_*to*_, *I*_*k1*_, *I*_*kur*_, and *I*_*kATP*_. Aconitine acted on both *I*_*kur*_ and *I*_*k1*_ potassium channel inhibition (Wang et al., 2008; Kiss et al., 2017). Aconitine also could cause changes in *I*_*to*_ current channel of rat cardiomyocytes by reducing voltage-gated potassium channel 4.3 (Kv4.3) mRNA expression (Dong et al., 2009). Celastrol has a dual effect on *I*_*k1*_ channel, including blocking potassium channel and inhibiting channel protein expression on cell surface (Sun et al., 2006). As_2_O_3_ activated *I*_*kATP*_ and inhibited *I*_*k1*_ *via* upregulating miR-1 expression (Shan et al., 2013).

#### Calcium Homeostasis

Calcium is one of the most ubiquitous signal transduction molecules, which mediates various biological functions, such as cell excitability and conductivity, excitation–contraction coupling, and programmed cell death (Landstrom et al., 2017). Intracellular calcium homeostasis involves a dynamic balance between intracellular calcium intake and calcium emission. Abnormality of L-type Ca^2+^ currents (*I*_*Ca-L*_), ryanodine receptor (RyR), sarcoplasmic reticulum Ca^2+^-ATPase (SERCA), and sodium–calcium exchanger (NCX) disrupts calcium homeostasis. Cardiac calcium homeostasis disorder contributes to adverse reactions induced by CMM. *I*_*Ca-L*_ is activated upon membrane depolarization, and then Ca^2+^ influx triggers the release of Ca^2+^ *via* calcium release channels in sarcoplasmic reticulum (SR) (Bito et al., 2008; Eisner et al., 2009). Therefore, disturbance of *I*_*Ca-L*_ channel can lead to fatal cardiac arrhythmias (Eden et al., 2016). Bradycardia and hypotension caused by improper use of aconite may be attributed to the inhibition of *I*_*Ca-L*_ channels (Chou et al., 2018). Aconitine could block *I*_*Ca-L*_ channels and calcium-induced calcium release (CICR) in human-induced pluripotent stem cell–derived cardiomyocytes (hiPSC-CMs) (Wu et al., 2017). RyR is a calcium-release channel on ER/SR, which also plays a crucial role in maintaining intracellular calcium concentration. The sensitivity changes of RyR2 to Ca^2+^ release activation have been implicated in arrhythmias and heart failure (Dobrev and Wehrens, 2014; Landstrom et al., 2017). RyR2 activation may have a direct relationship with aconitine-induced arrhythmias (Fu et al., 2008). A study showed that aconitine significantly increased mRNA and protein expressions of RyR2 and NCX, thereby leading to uncoupling of Ca^2+^ signaling (Scriven et al., 2000; Bers, 2002). Oleander extract might markedly increase calcium concentration by inhibiting RyR in cardiomyocytes (Poindexter et al., 2007). RyR activity can be regulated by calmodulin (CaM) or calmodulin kinase (CaMK). For example, hypaconitine could downregulate CaM mRNA and protein expression, thus inhibiting CaMK II activation and RyR2 phosphorylation (Yi et al., 2012). Furthermore, CaM inhibits the opening of calcium release channel by binding to RyR2 (Sigalas et al., 2009a; Sigalas et al., 2009b). Intracellular Ca^2+^ diffusion is highly restricted due to the elaborate cytosolic Ca^2+^ chelating machinery. Intracellular calcium is mainly excreted through NCX and SERCA2a (Muller, 2018). NCX plays a pivotal role in regulating contractility and electrical activity in the heart. Studies found that arrhythmogenesis toxicity of aconitine was related to the increased expression of NCX (Fu et al., 2007; Zhou et al., 2013). NCX can operate in both Ca^2+^ efflux and influx modes depending on the internal and external concentrations of Na^+^ and Ca^2+^. Therefore, disturbance of NCX and SERCA function may destroy cytoplasmic calcium ion gradient, which not only induces calcium overload but also hinders the rapid entry of calcium ions into the cytosol after the ion channel is opened. Multiple studies have demonstrated that Ca^2+^ overload leads to oxidative damage and ER stress (Dong et al., 2010; Groenendyk et al., 2010; Chao et al., 2012; Guo et al., 2013; Xie et al., 2015). As_2_O_3_ inhibited SERCA2a activity in a time-dependent manner, thereby triggering ER stress (Zhang et al., 2016a). Aconitine-induced cardiotoxicity is related to intracellular Ca^2+^ elevation by accelerating *I*_*Ca-L*_, increasing NCX expression, and decreasing the SERCA2a level (Zhou et al., 2013). Intracellular calcium homeostasis imbalance is one of the most important mechanisms underlying CMM-induced cardiotoxicity.

#### Ion Transport–Related ATPases

Ion transport–related ATPases also contribute to the cardiotoxicity of CMM. Sodium–potassium ATPase (Na^+^-K^+^ ATPase) maintains intracellular potassium and sodium ion gradients *via* consuming ATP. The steep sodium and potassium gradients in animal cells not only contribute to the rapid signal transmission through the opening of sodium or potassium selective channels in the plasma membrane in response to extracellular signals but also are used to facilitate the secondary transport of sugars, neurotransmitters, amino acids, metabolites, and some ions, such as H^+^, Ca^2+^, and Cl^−^ (Clausen et al., 2017). Egyptian green toad *Bufo viridis* skin secretions and bufalin inhibited the Na^+^-K^+^ ATPase activity, thus inducing cardiac adverse reaction (Roger et al., 2002; Abdel-Rahman et al., 2010). Ca^2+^-ATPase and Ca^2+^-Mg^2+^ ATPase can promote the removal of Ca^2+^ from cell membrane after myocardial contraction. Once the activities of these Ca^2+^-ATPases decreased, intracellular Ca^2+^ continuously increased to Ca^2+^ overload. Aconitine could cause calcium overload, which involved the inhibition of Na^+^-K^+^ ATPase, Ca^2+^-ATPase, and Ca^2+^-Mg^2+^ ATPase activities (Zhang et al., 2016b).

### Oxidative Stress

Oxidative stress is a result of overproduction of oxidative-free radicals and ROS (Newsholme et al., 2016). Excessive oxidative stress not only directly causes DNA and protein damage but also induces lipid peroxidation, calcium overload, mitochondrial structure and function damage, and cell death, thereby inducing myocardial damage (Manna et al., 2008; Zhang et al., 2013a; Xu et al., 2014; Yang et al., 2017). Previous studies found that triptolide increased intracellular ROS, which in turn depolarized mitochondrial membrane potential (MMP), reduced the Bax/Bcl-2 ratio, released cytochrome C, activated caspase-3, and ultimately triggered cell apoptosis (Zhou et al., 2014). Similarly, evodiamine also increased intracellular ROS level, induced lipid peroxidation, and caused changes in cell membrane stability, ultimately resulting in myocardial damage (Yang et al., 2017). Oxidative stress also can trigger the inflammatory reaction and inhibit ATPase activity. For example, ROS induced by As_2_O_3_ was able to activate pro-inflammatory genes and upregulate the expression of inflammatory cytokines, leading to the occurrence of inflammatory reaction in myocardium. As_2_O_3_ also inhibited the ROS-mediated myocardial membrane ATPase activity and disrupted the steady state of intracellular electrolytes (Binu et al., 2017; Li et al., 2017). Moreover, calcium mobilization can affect ROS generation, and on the contrary, the presence of ROS induces Ca2+ influx (Giambelluca and Gende, 2008; Holmstrom and Finkel, 2014). ROS can regulate calcium pump, enzyme, and receptor activities to maintain normal physiological function of cardiomyocytes (Feissner et al., 2009). Additionally, ROS impairs mitochondrial structure and function both alone and in combination with calcium overload (Webster, 2012). Mitochondria permeability transition pore (mPTP) is sensitive to both ROS and calcium overload, thereby triggering endogenous pathways of programmed necrosis and death.

Cardiomyocytes can activate an antioxidative stress cell defense system to resist myocardial damage caused by oxidative stress. However, long-term or excessive use of CMM that can induce cardiotoxicity will destroy the antioxidant defense system and further aggravate oxidative stress. For example, As_2_O_3_ reduced GSH-dependent antioxidant and anti-peroxidase enzymes, while increasing free radicals, intracellular Ca^2+^ concentration, and lipid peroxidation, thereby further exacerbating oxidative damage (Sfaxi et al., 2016; Varghese et al., 2017). Another study also showed that As_2_O_3_ induced ROS generation and downregulated the expression of Nrf2 and heme oxidase (HO-1) genes (Zhang et al., 2013a).

### Mitochondrial Toxicity

Mitochondria are one of the most critical target organs for cardiotoxicity induced by CMM. Some CMM can directly damage mitochondrial structure and function. For example, As_2_O_3_ promoted mitochondrial peroxide production; significantly reduced the activity of respiratory chain complexes, such as mitochondrial complexes I, III, and IV; reduced MMP; and caused mitochondrial swelling in H9c2 cardiomyocytes (Vineetha et al., 2015). Mitochondrial dysfunction is characterized by an increase in mitochondrial outer membrane permeabilization (MOMP), which induces mitochondrial swelling following proapoptotic protein release, electron transport chain disruption, and ATP depletion (Zhang et al., 2018a). The opening of mPTP contributes to MOMP changes *via* the depletion of MMP. The persistent opening of mPTP is now recognized as a major inducement of cell death. Triptolide inhibited superoxide dismutase (SOD) and catalase (CAT) activities, induced ROS accumulation, reduced MMP, and eventually led to mitochondrial toxicity (Yang et al., 2016). Experiments *in vivo* and *in vitro* showed that triptolide permeabilized the outer mitochondrial membrane and promoted the opening of mPTP, thereby causing mitochondrial dysfunction and apoptosis (Wang et al., 2016b; Xi et al., 2018). In addition, CMM that has cardiotoxicity can also indirectly damage myocardial mitochondria by regulating mitochondrial outer membrane integrity, mitochondrial dynamics, mitochondria-dependent apoptosis genes, and proteins. For instance, triptolide triggered MOMP through the p53-regulated Bcl-2 family, which elicited mitochondria dysfunction and apoptosis (Xi et al., 2018). As_2_O_3_ reduced peroxisome proliferator-activated receptor gamma coactivator 1 (PGC-1α) level, and disrupted mitochondrial synthesis and fission and fusion function (Di et al., 2018).

### Induction of Cell Death

Oxidative stress and calcium overload are the main inducements for triggering programmed cell death in cardiomyocytes. As mentioned above, long-term unreasonable use of cardiotoxic CMM can induce ROS generation and disturb calcium homeostasis. One study showed that As_2_O_3_ could initiate ER stress apoptosis pathway through ATF6, PERK, and IRE1 (Zhang et al., 2017). As an active programmed cell death pathway, apoptosis is regulated by multiple signaling pathways. Aconitine could upregulate the expression of p38 MAPKs, further damage MMP, induce changes in mitochondrial permeability, activate caspase-3, and ultimately result in mitochondria-dependent apoptosis (Sun et al., 2014). The p53 signaling pathway plays an important regulatory role in triptolide-induced apoptosis (Xi et al., 2018). Triptolide downregulated the expression of Nrf2 and induced the ROS-triggered mitochondrial apoptosis pathway (Vineetha et al., 2018). The serine/threonine kinase Akt signaling pathway was also found to be involved in the regulation of As_2_O_3_-induced mitochondrial apoptosis in cardiomyocytes (Wang et al., 2013). Autophagic degradation of cellular components is essential to maintain cellular homeostasis. Hence, autophagy, another type of programmed cell death, contributes to cardiotoxicity induced by CMM. Excessive release of ROS is one of the main endogenous factors to enhance autophagy (Scherz-Shouval et al., 2007). As_2_O_3_ induced autophagy and apoptosis by promoting oxidative stress (Fan et al., 2014). The signaling pathways currently known to participate in the regulation of autophagy comprise Parkin, ROCK, PI3K/Akt/mTOR, p70S6 kinase, and ERK1/2 (Huang et al., 2015; Zhou et al., 2015). Another study showed that As_2_O_3_ induced a perturbation in Parkin-mediated mitophagy (Watanabe et al., 2014). Besides, arsenic induced cardiac damage and autophagy *via* activating the PI3K/Akt/mTOR pathway in the hearts of chickens (Li et al., 2018b). Excessive autophagy was susceptible to various cardiovascular events, such as ischemia-reperfusion injury (Zheng and Bao, 2017). Autophagy can suppress apoptosis under normal physiological conditions. For example, aconitine induced autophagy and apoptosis, in which autophagy had an antagonistic effect on apoptosis (Hu et al., 2018). Whether autophagy is beneficial or detrimental depends on intracellular autophagic machinery and the burden of substrate receptor proteins targeted for autophagy (Baehrecke, 2005).

### Involvement of Substance Metabolism

#### Amino Acid Metabolism

Amino acid metabolism affects protein synthesis, energy metabolism, redox balance, and intracellular material homeostasis. Metabolic disorders of various amino acids, such as tryptophan, leucine, alanine, proline, lysine, and tyrosine, were involved in CMM-induced cardiotoxicity. Tryptophan plays a vital role in maintaining cell growth and energy metabolism. In metabolomic studies of some CMM, such as *Radix Aconiti Kusnezoffii* (Caowu), *Radix Aconiti Lateralis Preparata* (Fuzi), *Pinellia ternata*, and *Periploca sepium Bunge*, tryptophan significantly reduced in myocardial cells, which affected energy supply to produce cardiotoxicity (Wang et al., 2012; Zhang et al., 2013c; Li et al., 2016a). In addition, experimental results showed that valine, leucine, and proline all decreased in cardiomyocytes exposed to periplocin, suggesting that amino acid metabolism disorders occurred during periplocin-induced cardiomyocyte toxicity (Li et al., 2016a). Lysine, alanine, and arginine in serum of *Venenum Bufonis*–treated rats showed a decreasing trend, indicating that oxidative stress injury and energy metabolism disturbance were induced by *Venenum Bufonis* (Dong et al., 2015). Different from the above amino acids, l-carnitine is a type of nonprotein amino acid. Carnitine and acetylcarnitine are also involved in fatty acid transport and oxidation by mitochondrial enzymes, which is essential for the formation of acetyl-CoA (Reuter and Evans, 2012; Li et al., 2015). Furthermore, l-carnitine also has antioxidant activity to improve mitochondrial function. Periplocin significantly reduced carnitine and acetylcarnitine in myocardial tissues, revealing that tricarboxylic acid (TCA) cycle related with energy metabolism was interfered (Li et al., 2016a). On the contrary, both l-carnitine and l-acetylcarnitine significantly increased after *Pinelliae Rhizoma* administration (Zhang et al., 2013c).

#### Lipid Metabolism

Fatty acids and their derivatives are involved in saturated fatty acid oxidation and unsaturated fatty acid peroxidation. β-oxidation of fatty acids, an important pathway for mitochondrial function in cardiomyocytes, is closely related to glucose metabolism and apoptosis in cardiomyocytes. In myocardial lipidomic study of Fuzi, arachidonic acid, linoleic acid, and eicosapentaenoic acid displayed declining tendencies, while saturated fatty acid levels, such as stearic acid and 5-tridecynoic acid, were upgraded. Peroxidation of unsaturated fatty acids produces active free radicals that induce oxidative stress. Accordingly, abnormal peroxidation of unsaturated fatty acids caused by Fuzi contributed to oxidation damage in myocardial mitochondria (Cai et al., 2013). Similarly, oleic acid and linoleic acid were downregulated, while arachidonic acid was upregulated in the metabolomic study of *Venenum Bufonis*. *Venenum Bufonis* could interfere with lipid metabolism through the inhibition of free fatty acid reacylation or the activation of protein kinase pathways, thereby inducing heart damage (Liang et al., 2011).

As a critical component of cellular membranes, phospholipids interact with all membrane proteins and various non-membrane proteins, thus mediating signal transduction. Significant changes of sphingosine, phosphatidylcholine, phosphatidylserine, phosphatidylethanolamine, lysophosphatidylethanolamine, and lysophosphatidylcholine were uncovered in CMM-induced cardiotoxicity. These substances are involved in metabolic pathways, such as phospholipid and sphingolipid metabolism. For example, Caowu- and Fuzi-induced cardiotoxicity were responsible for sphingolipid metabolism (Wang et al., 2012). Sphingolipids have emerged as key mediators of stress responses (An et al., 2011). Sphingolipid metabolism disorder resulted in increased plasma-membrane fluidity, which rendered them susceptible to ROS, thus enhancing oxidative damage to cellular proteins (Rao et al., 2007). Studies showed that perturbation of phospholipid or sphingolipid metabolism contributed to *Pinellia ternate* and periplocin-induced cardiotoxicity (Zhang et al., 2013c; Li et al., 2016a).

#### Glucose Metabolism

Abnormal blood glucose is an important trigger for cardiovascular diseases. The anaerobic glycolysis and aerobic oxidation of glucose in metabolism pathway are the main link for ATP production. Metabolomic studies showed that various cardiotoxic CMM induced glucose metabolism disturbance, which mainly involved some metabolites changes, such as pyruvic acid, malic acid, citric acid, and succinate (Li et al., 2008; Li et al., 2016a). These organic acids as an important intermediate of cell cycle affect TCA cycle of myocardial cells and energy metabolism. In addition, metabolomic analysis of aconitum alkaloids showed that lactate and lipids levels increased, while glucose reduced in myocardial cells, demonstrating that aconitum alkaloids induced abnormality in energy utilization with a higher glycolysis rate (Sun et al., 2009).

### Other Mechanisms

DNA damage, inflammatory response, and trace element homeostasis imbalance may also be involved in CMM-induced cardiotoxicity. For instance, berberine and arsenic could induce DNA damage in heart cells (Manna et al., 2008; Xu et al., 2014). Triptolide inhibited RNA polymerase by covalently binding to a subunit of transcription initiation factor TFIIH, which led to the inhibition of nucleotide excision repair and DNA damage (Titov et al., 2011). *Venenum Bufonis* inhibited the expression of Fxyd domain-containing ion transport regulator 3 (Fxyd3) and ceruloplasmin (Cp) genes, which resulted in the accumulation of Fe^2+^ in cardiac cells to destroy contractile function of the heart (Yang et al., 2011a). Recently, inflammatory response plays a critical role in the development, course, severity, and outcomes of heart failure (Papadimitriou and Kalogeropoulos, 2015). As_2_O_3_ and aristolochic acid could trigger inflammatory response, which was related to the progressive development of cardiac defects (Li et al., 2017; Shi et al., 2017). A study on the cardiotoxicity of aristolochic acid in zebrafish embryos revealed that a number of pro-inflammatory genes increased, including COX-2, IL-1β, C/EBPB, C/EBPG, and SAA (Huang et al., 2007; Shi et al., 2017). *Venenum Bufonis*–induced cardiotoxicity might involve inflammatory response through TXNIP/TRX/NF-κB and MAPK/NF-κB pathways (Bi et al., 2016). In addition, As_2_O_3_ was reported to disturb trace elements’ homeostasis, which favored the progression of mitochondrial damage (Sfaxi et al., 2016; Li et al., 2018a).

## Discussion

With the booming market of CMM worldwide, a new evaluation system must be formulated for assessing drug efficacy, effectiveness, and toxicity to optimize the therapeutic and preventive potential of CMM. In recent years, the safety of CMM has been paid more and more attention by clinicians, patients, and scientific researchers, such as the cardiotoxicity of aconitum species, the hepatotoxicity of *Polygonum multiflorum*, and the nephrotoxicity of aristolochic acids. Some CMM have been proven to present obvious cardiotoxicity in literature records, case reports, and experimental studies. In response to the problem of adverse cardiac reactions induced by CMM, research teams have carried out a large number of studies on the performances and characteristics, toxic substance basis, and mechanisms of cardiotoxicity induced by CMM, their extracts, or single compounds. Our review concluded the risk compounds, preclinical toxicity evaluation, and potential mechanisms of CMM-induced cardiotoxicity, which was beneficial to establish a preclinical evaluation system for CMM safety to promote the development of new drugs.

CMM has a complex source, numerous varieties, and a wide variety of phytochemical components. Most of CMM have substances with toxicity and efficacy dual characters, such as aconitine, bufotoxin, and cardiac glycosides (Peng, 2017; Peng et al., 2017). Furthermore, toxic compounds and active compounds containing the same type of CMM can be transformed into each other under different physiological and pathological conditions. For example, diester alkaloids derived from aconitum species are considered highly toxic substances, while they have also proved to be the main active ingredient for the treatment of pain and leukemia (Nie et al., 2017). In addition, chemical composition and content may change under different planting resources, processing methods, or drug compatibility. Thus, more and more researchers select risk compounds in CMM as the research objects to explore the mechanisms of CMM-induced cardiotoxicity. With the advancement of modern separation and analysis technologies, the types and quantities of monomer compounds in traditional toxic CMM are increasing. High-throughput analysis methods, such as omic technology, high content analysis, molecular docking, and network pharmacology, are used to determine the risk substances of CMM-induced cardiotoxicity (Shi et al., 2016; Su et al., 2016; He et al., 2019). Based on the phytochemical structures of toxic substances in CMM, these risk compounds mainly include alkaloids, terpenoids, steroids, heavy metals, organic acids, and toxic proteins or peptides. Alkaloids are the biggest category of risk compounds present in CMM that induce cardiotoxicity, including diterpene alkaloids, isoquinoline alkaloids, indole alkaloids, and others. Terpenoids are also an important substance of CMM-induced cardiotoxicity, such as triptolide, celastrol, and rhodojaponin. Various steroids, including cardiac glycosides, bufadienolides, and ophiopogonin, also contribute to CMM-induced cardiotoxicity. In addition, heavy metals, organic acids, toxic proteins, and peptides are involved in CMM-induced cardiotoxicity. Phytomedicine is easily affected by environmental pollution, thus inducing heavy metals to exceed the standard, and mineral drugs contain high levels of heavy metals, such as arsenic, mercury, and lead. Toxic plants and animals carry or secrete organic acids, toxic proteins, or peptides to defense natural enemies.

Multicomponent, multi-target, and multichannel characteristics of CMM make the toxicological mechanism very complicated. Most of the previous studies focus on single compound, single target, or single pathway to explore the mechanism of CMM-induced cardiotoxicity. With the development of various modern biological techniques, such as systematic toxicology, network toxicology, and computer toxicology, the network interaction mechanism in CMM-induced cardiotoxicity is gradually uncovered. We searched the related literature for the latest twenty years, mainly focusing on cell and animal experiments. Various mechanisms, including ion homeostasis, oxidative stress, mitochondrial damage, apoptosis and autophagy, and metabolic disturbance, are involved in CMM-induced cardiotoxicity. Excessive activation or inhibition of ion channels, including sodium, potassium, and calcium channels, contributes to arrhythmia. Ion channels can become the targets for many toxic CMM, such as aconitine, hypaconitine, berberine, and *Venenum Bufonis*. Therefore, imbalance among sodium, potassium, and calcium ions is the important mechanism of CMM-induced cardiotoxicity. Overproduction of ROS resulting in oxidative damage has been testified to be the vital factor for calcium overload, which suggested that oxidative damage affected intracellular calcium homeostasis. Mitochondria are highly susceptible to ROS generation. The risk substances in CMM can directly or indirectly influence mitochondrial structure and function, thus presenting mitochondrial toxicity. Both oxidative stress and abnormal ion homeostasis lead to excessive apoptosis and autophagy in cardiomyocytes. Apoptosis and autophagy-related genes or pathways may be activated or inhibited to regulate cell fate. Abnormal metabolism in cardiac cells also results in cardiotoxicity. Mitochondria toxicity induced by the risk compounds in CMM can affect cell energy metabolism. In addition, substance metabolism, including amino acid, lipids, and glucose, was also reported to be involved in the cardiotoxicity of CMM. Thus, multiple mechanisms may be orderly involved in the cardiotoxicity induced by CMM, and an interactive effect between these mechanisms is demonstrated. Although the material basis of CMM-induced toxicity and efficacy can be transformed into each other under certain conditions, researches on the dosage, opportunity, and body status of transformation mechanism between the toxicity and efficacy are still scarce. Therefore, the toxicity–efficacy transformation mechanism underlying the “dose–time–efficacy/toxicity” path is urgent to be clarified for evaluating the cardiotoxicity of CMM (Peng et al., 2008; Peng, 2017; Peng et al., 2017).

In clinical studies, there are few specific evaluation indicators for CMM-induced cardiotoxicity. At present, evaluation on CMM-induced cardiotoxicity mainly takes the indicators of cardiac function and structural damage caused by chemical drugs as a reference, including ECG, myocardial zymogram, echocardiography, endomyocardial biopsy, and biomarkers. In preclinical evaluation or new drug development stage, the cardiotoxicity of CMM should be systematically evaluated at the overall, tissue, cellular, and molecular levels. Overall evaluation is mainly based on cardiac function evaluation, including conventional ECG, echocardiography, and MRI, which is especially manifested with QT/QTc interval prolongation or TdP. Comprehensive study of QT/QTc is the most critical link of drug cardiac safety evaluation (Food and Drug Administration, 2005). Observation on the histopathological changes of the heart caused by CMM is one of the specific diagnostic criteria. For example, although endomyocardial biopsy technique is recognized as the most sensitive and specific method for evaluating cardiac toxicity induced by drugs, it is invasive examination with high technical requirements (Jones and Miles, 2005). At the cellular level, it is possible to observe changes in cell morphology, myocardial cell pulsation, cell survival rate, and cell membrane integrity, which are of great significance for revealing the mechanism of drug-induced cardiotoxicity. Serum myocardial zymogram, including CK, CK-MB, LDH, and AST, may be slightly different because of different test kits. Due to the poor specificity of LDH, AST, and CK for assessing cardiotoxicity, CK-MB is the commonly used clinical myocardial enzyme. Drug-induced heart injury can lead to many biomarkers changes in the early stage, including proteins or polypeptides, metabolic small molecules, and genes. Common proteins or peptides include cardiac troponins, BNP, NT-proBNP, ANP, and H-FABP. cTnT/TnI can detect early cardiotoxicity caused by anthracyclines. BNP and NT-proBNP can objectively determine the severity of heart failure. H-FABP is the most ideal myocardial marker for identifying myocardial injury. Small metabolic molecules include citric acid, GSH, phosphatidylcholine, and uric acid in serum. Metabolomic method is expected to find more specific small metabolic molecules for evaluating CMM-induced cardiotoxicity (Su et al., 2016; Liu et al., 2019). In addition, genes related to cardiotoxicity are also used to early identify the cardiotoxicity of CMM, such as transcription factors, inflammatory factors, pathway regulatory genes, and microRNAs. MicroRNAs have potential as early biomarkers and diagnostic tools for cardiotoxicity (Skala et al., 2019a).

## Conclusion

Numerous reports about drug safety have brought more cautions that CMM is associated with several cardiovascular events, suggesting that evaluation on the cardiac safety of CMM is quite urgent. CMM-induced cardiotoxicity is characterized by diverse components, multiple manifestations, and complex mechanisms. Thus, we concluded the risk compounds, preclinical toxicity evaluation, and potential mechanisms of CMM-induced cardiotoxicity through screening the related studies for the last twenty years. It is worth noting that a complex network regulation relationship among these risk compounds, toxic effects, and toxic mechanisms is being gradually revealed. Frontier technologies, such as omics, systematic toxicology, and network pharmacology, provide important support for revealing this network interaction relationship. Our review is greatly beneficial to understand the relationship between the toxicity and efficacy induced by CMM and provide a reference for establishing the cardiac safety evaluation system of CMM.

## ABBREVIATIONS

CMM: Chinese materia medica
ECG: Electrocardiogram
ICH: International Conference on Harmonisation
CNKI: China National Knowledge Infrastructure
hERG: Human ether-a-go-go-related gene
HR: Heart rate
MRI: Magnetic resonance imaging
APD: Action potential duration
TdP: Torsades de pointes
LVEF: Left ventricular ejection fraction
BMSCs: Bone marrow mesenchymal stem cells
CK: Creatine kinases
CK-MB: Creatine kinase isoenzymes
LDH: Lactic dehydrogenase
AST: Aspartate aminotransferases
α-HBDH: α-Hydroxybutyrate acid dehydrogenases
BNP: Brain natriuretic peptide
NT-proBNP: N-terminal brain natriuretic peptide precursor
ANP: Atrial natriuretic peptide
cTnT: Troponin T
cTnI: Troponin I
FABP: Fatty acid–binding protein
GSH: Glutathione
ROS: Reactive oxygen species
1HNMR: Nuclear magnetic resonance hydrogen spectroscopy
Fxyd3: Fxyd domain-containing ion transport regulator 3: Fxyd domain-containing ion transport regulator 3
Rnd1: Rho family GTPase 1
AMI: Acute myocardial infarction
VGSCs: Voltage-gated sodium channels
Sp1: Specificity protein 1
UPR: Unfolded protein response
ER: Endoplasmic reticulum
Kv4.3: Voltage-gated potassium channel 4.3
ICa-L: L-type Ca2+ currents
RyR: Ryanodine receptor
SERCA: Sarcoplasmic reticulum Ca2+-ATPase
NCX: Sodium–calcium exchanger
SR: Sarcoplasmic reticulum
CICR: Calcium-induced calcium release
hiPSC-CMs: Human-induced pluripotent stem cell–derived cardiomyocytes
CaM: Calmodulin
CaMK: Calmodulin kinase
MMP: Mitochondrial membrane potential
mPTP: Mitochondria permeability transition pore
HO-1: Heme oxidase
MOMP: Mitochondrial outer membrane permeabilization
PGC-1α: Peroxisome proliferator-activated receptor gamma coactivator 1
TCA: Tricarboxylic acid
Fxyd3: Fxyd domain-containing ion transport regulator 3: Fxyd domain-containing ion transport regulator 3
Cp: Ceruloplasmin
